# Bioinspired protocells exhibiting anti-phytopathogenic properties

**DOI:** 10.3389/fmicb.2025.1741951

**Published:** 2026-01-09

**Authors:** Walter Riofrío

**Affiliations:** Departamento de Estadística, Demografía, Humanidades y Ciencias Sociales, Facultad de Ciencias e Ingeniería, Universidad Peruana Cayetano Heredia, Lima, Peru

**Keywords:** biomimicry, compartmentalization, nanosponges-hydrogel, phytopathogens, proteasome, protocells, reactive oxygen species, signaling network

## 1 Introduction

The outcome of infectious diseases is generally believed to be influenced by the interactions between the host and the pathogen. Moreover, these exchanges occur over a sequence of phases or stages (Duneau and Ferdy, 2022). A sequence of molecular signal transmissions on the host side stimulates the plant's intrinsic immune system in response to pathogens, leading to the establishment of disease resistance mechanisms (Andersen et al., 2018; Ding et al., 2022; Khan et al., 2023).

The two main layers of plant pathogen defense are PTI and ETI. PTI is a basic immune response that uses pattern-recognition receptors to identify conserved microbial entities. Plants use membrane-bound pattern recognition receptors (PRRs) to perceive microbe- or pathogen-associated molecular patterns (MAMPs or PAMPs) to activate basal immunity, known as PAMP-triggered immunity. When intracellular resistance proteins (R-proteins) recognize pathogen effectors, which infections use to weaken host defenses, ETI is a powerful and precise response. ETI initiates a quick and vigorous defense response, sometimes with a hypersensitive reaction (HR) that confines the pathogen, providing stronger and longer-lasting resistance than PTI (Zhang et al., 2010; Nguyen et al., 2021). The plant's immune signaling network involves the exchange and recognition of a complex, generalized set of signals. Furthermore, intercellular communication is crucial for plant defense, and it occurs primarily thru apoplastic and symplastic pathways (Tabassum and Blilou, 2022).

Conversely, phytopathogens have developed mechanisms to adeptly alter the host's cellular and hormonal signals during the plant–pathogen relationship. Many specific molecular processes implicated in phytopathogenic infections remain inadequately elucidated (Li et al., 2022).

We don't understand the order and timing of all plant pathogen-induced infection phases. However, we know the particular interactions that start an infection. This opinion article will concentrate our inquiry on the initial moment of interaction between the host and the infectious agent: the apoplast.

## 2 The apoplast: the first immune defense

A significant portion of a plant's immune response consists of intracellular processes, including transcriptional reprogramming and MAPK signaling (Taj et al., 2010; Plotnikov et al., 2011; Zhong et al., 2023). Nonetheless, we are starting to comprehend that the majority of plant-pathogen interactions happen in the extracellular region referred to as the apoplast. Moreover, if the infection were effective, it would serve as a significant locus for pathogen proliferation (Jones and Dangl, 2006; Doehlemann and Hemetsberger, 2013; Jashni et al., 2015).

The apoplast comprises the non-living elements of a plant's extracellular space, including cell walls, intercellular spaces, and xylem vessels. The components create a continuous network outside the plasma membrane, thereby playing crucial roles in water transport, nutrient absorption, and plant defense signaling (Farvardin et al., 2020; Dora et al., 2022). Our analysis underscores the apoplast as the primary locus of interaction between plants and pathogens, wherein the chemicals contained inside contribute to plant defense mechanisms (Godson and van der Hoorn, 2021). This final assertion is crucial to our research, as the apoplast serves as the initial barrier against invading viral, bacterial, and fungal pathogens (Farvardin et al., 2020; Darino et al., 2022).

### 2.1 The apoplast and its proteasome

Plant proteases are crucial in nearly all facets of plant life. Plant genomes contain several proteases that play roles in development, homeostasis, biotic and abiotic stress responses, symbiosis, and growth (van der Hoorn and Klemenčič, 2021).

Considering the significance of proteases, it is essential to acknowledge that the apoplast, the plant's extracellular matrix, is the initial domain that plants must protect against invading pathogens. Consequently, new research findings indicate that it encompasses a substantial quantity of proteases, which may play a role in immunity.

Godson and van der Hoorn (2021) conduct a meta-analysis of 46 apoplastic proteases implicated in plant immunity, examining their localization, functionality and, its fundamental role in the plant immune responses. Most apoplastic immune proteases are found to be generated in response to pathogen infection, with 17 being genetically essential for the plant immune response. The analysis finds six separate methods by which these proteases facilitate plant defense and addresses the obstacles for future research.

But we also have to mention the existence of a large protein complex: the proteasome. A proteasome in plants is a large and essential protein complex for the selective degradation of damaged, unnecessary, or misfolded proteins. It is part of the ubiquitin-proteasome system, which marks proteins for breakdown into peptides and amino acids, thus enabling the recycling and regulation of proteins key to cellular function, development, and response to stress, such as pathogen attack (Kurepa et al., 2009; Mackinnon and Stone, 2022; Langin et al., 2023).

A study of *Arabidopsis* found active proteasomes in the plant apoplast. This suggests that “exoproteasomes” degrade pathogen-released proteins to aid the plant's immunological response. An innovative method for enriching *Arabidopsis* plant apoplastic fluid was used to make the discovery. To confirm proteasome presence and activity, microscopic detection, proteasome-specific activity assays, immunological assays, and mass spectrometry were used. Thus, the study found that these extracellular proteasomes (exoproteasomes) are active in the apoplast and help to promote basal pathogen defense. They do this by cleaving bacterial proteins, such as flg22 from flagellin, which are associated with molecular patterns (MAMPs or PAMPs) that trigger a plant immune response. The findings also suggested that pathogens have the ability to bypass this response. The interaction between plants and pathogens was exemplified by syringolin-A, a virulence effector from Pseudomonas syringae that inhibited exoproteasome activity (Karimi et al., 2025).

### 2.2 ROS bursts after perception of pathogens

Reactive oxygen species (ROS) are oxygen-containing reactive molecules, including superoxide (·${\text{O}}_{2}^{-}$), hydrogen peroxide (H_2_O_2_), and hydroxyl radical (·OH) (Mori and Schroeder, 2004; Sagi and Fluhr, 2006).

Extensive evidence indicates that ROS function as cellular signaling molecules, facilitating critical responses of plant cells to many physiological stimuli, such as pathogen invasion, abiotic stress, hormonal signaling, and polar growth (Torres and Dangl, 2005).

It has been suggested that biomembrane channels (aquaporins) facilitate H2O2 transport across biological membranes to regulate ROS signaling in plant cells (Bienert and Chaumont, 2014; Tian et al., 2016).

The primary ROS bursts upon pathogen detection occur in the apoplast; yet, ROS generated in several intracellular compartments may play a role in the plant's defense against pathogen invasion (Torres, 2010).

ROS serve as a potent tool that can be swiftly generated and deployed in the fight against pathogen infection. These substances are released from the cell within 3 min following the recognition of MAMP or PAMP (Chinchilla et al., 2007; Nühse et al., 2007; Hedrich, 2012).

The recognition of PAMPs by plants through PRRs initiates the generation of ROS through the activation of NADPH oxidases and peroxidases, resulting in PTI-dependent basal defenses that impede invading pathogens. The apoplastic ROS bursts produced in elicited plant cells possess a level of cytotoxicity capable of eliminating invading pathogens (Legendre et al., 1993; Chi et al., 2009; Park et al., 2013).

ROS function as signaling molecules, initiating immune responses and cell death in plants (Tenhaken et al., 1995; Jabs, 1999; Torres, 2010). In light of this, pathogens are required to devise methods that will allow them to avoid being exposed to potentially hazardous reactive oxygen species. In the event that plants are infected with a pathogen, NADPH oxidases, which are also known as respiratory burst oxidase homologs (RBOHs), play a significant part in the formation of ROS (Torres and Dangl, 2005).

## 3 Biomimicry of cellular defense against pathogens

The primary defense against pathogen invasion in plants occurs in the apoplast via bursts of ROS, they serve as a formidable defense against the initial assault of a pathogen and represent a swift element of cellular signaling that activates immunological responses (Tenhaken et al., 1995; Jabs, 1999; Torres, 2010).

Consequently, based on the information derived from research on pathogen infections, we can formulate specific arguments that can be inferred from the preceding discussion. These arguments would finally seek to secure plausible alternatives that could strengthen this first line of defense.

It can be established that ROS bursts in the apoplast are highly effective in mitigating or diminishing pathogen infection levels in plants. Consequently, numerous pathogens are recognized to have evolved mechanisms that inhibit or suppress the production of reactive oxygen species in the apoplast (Daudi et al., 2012).

We succinctly outline how pathogens inhibit the apoplastic ROS burst below. Certain pathogens, including biotrophic fungi, can actively inhibit the plant's primary immune response (PAMP- or PTI-triggered immunity), hence inhibiting the initiation of the oxidative burst (Singh et al., 2021).

They can directly neutralize reactive oxygen species by synthesizing enzymes. Pathogenic fungi mitigate apoplastic ROS using many methods, including the synthesis of ROS-scavenging enzymes and antioxidant compounds. They also release extracellular effectors and inhibitors to neutralize or obstruct host ROS generation and their detrimental consequences, which are components of the plant's innate immune response (Chandrasekar et al., 2022; Hu et al., 2022).

Although other mechanisms exist, the aforementioned points suffice to assert that the apoplastic ROS burst serves as the primary defense that pathogens attempt to inhibit; thus, preserving or augmenting this first defense is inherently beneficial. There are two compelling explanations, the first being that it serves as the initial barrier against infection effort (Podgórska et al., 2017).

Furthermore, the elevated concentration of ROS in the apoplast activates several downstream reactions, such as modifications to the cell wall to regulate development and begin further defense mechanisms against pathogens (Kärkönen and Kuchitsu, 2015; Qi et al., 2017).

Due to emergent properties like molecular signaling, biological cells need compartmentalization to survive. Compartmentalization organizes cellular components into areas for precise signaling pathway regulation, and prevent crosstalk between pathways and ensure signals reach their targets (Arora et al., 2013; Condrea, 2023; Fayad et al., 2024).

In the bottom-up synthesis of cell-like entities, basic signaling mechanisms have been created to combine protocell behavior with cellular signaling networks, leveraging the fact that cell-to-cell communication via diffusible chemical signals is vital to life (Grimes et al., 2021).

In this context, as our conceptual idea aligns with bottom-up synthetic biology, we propose that an effective and logical answer would be the development of a bifunctional protocell. Initially, one that facilitates the preservation of ROS generation. Secondly, one capable of sequestering the toxins produced by the infection.

We propose to develop a dynamic biological system—a protocell—capable of generating ROS. Multiple methods exist to endow these protocells with a compartment dedicated to the generation of ROS (Xiong et al., 2019).

The second compartment of the protocell would comprise vesicles that transport pathogen toxins (possibly working with exoproteasomes). A viable strategy entails integrating vesicle-mediated transport within protocells to facilitate the passage of toxins from the external environment into the protocell. The technique involves the synthesis of nanostructures that exhibit high-affinity mimics of cellular receptors, preferentially binding to and neutralizing virulence factors (Bricarello et al., 2012), after which they are either sequestered or eliminated (Mason et al., 2019; Li et al., 2023).

Ultimately, both compartments will be incorporated into a nanosponges hydrogel. This formulation integrates nanosponges, solid, hypercrosslinked polymeric structures, within a hydrogel matrix for the controlled delivery of chemicals. This combination improves the stability of the nanosponges, maintains their localization, and facilitates the regulated release of molecules as well as the absorption of toxins or other active compounds (Wang et al., 2015).

## 4 Discussion

Proposing effective methods to enhance defenses in the apoplast seems to be a compelling alternative. Our theoretical construct proposal is based on the bottom–up approach of synthetic biology, aimed at designing a protocell with dual functionalities. The extracellular production of ROS primarily targets the pathogen. Secondly, the sequestration of certain poisons through their absorption into a compartment within the protocell.

The compartment responsible for ROS synthesis allows us to investigate whether its products interact with the ROS generated by RBOH enzymes, the primary source of ROS production in the apoplast. They would enhance the reactive oxygen species (ROS) generated by RBOHD, considered the central hub of ROS generation in the apoplast, thereby facilitating the propagation of the ROS wave and linking it to the Ca^2+^ wave to improve ROS transmission from localized to systemic tissues (Martínez Rivas et al., 2024).

Nanosponge technology is acknowledged for its capacity to absorb bacterial toxins by capturing and neutralizing them, hence obviating the need for unique manufacture for each type of toxin (Pashirova et al., 2023).
